# Dr. Prichard on Diseases of the Nervous System. Part I. Convulsive and Maniacal Affections

**Published:** 1822-06-01

**Authors:** 


### VIII.

*A Treatise on Diseases of the Nervous System. Part the First: comprising Convulsive and Maniacal Affections.* By J. C. Prichard, M. D. late of Trinity College, Oxford; Physician to St. Peter's Hospital and the British Infirmary. Octavo, pp. 427. London, 1822.

(First Analytical Article.)

Dr. Prichard's former works—his researches into the physical history of man; and his observations on fever have insured attention to every subsequent production from the same pen. We apprehend that the present work upholds the character of the writer, and will experience a favourable reception from the reading and thinking part of the profession. We say the reading *and* thinking—for there is but a comparatively small class who read *and* think. It unfortunately happens, too, that many of those works which have required the greatest thought in the construction, and demand a corresponding mental exertion in the perusal, are those which are least read or studied by the profession at large. We need hardly wonder at this, when we consider the harrassing labours of the practitioner, which must render him very much indisposed to the examination of books which impose the task of *study* after the fatigues of the day.

Can we wonder that this, which is the most numerous class, should gladly avail themselves of such analytical portraits as concentrate the pith or marrow of the subject, and thus lead to the direct application of principles at the bed-side of sickness? We have long had the most authentic means of knowing that, were it not for these analytical portraits, nine-tenths of our best productions would never travel beyond the circle of a few book-worms, or their information be diffused to any distance beyond the publisher's groaning shelves.

These reflections have been excited by a conviction that the valuable volume before us will never circulate, *in propria persona*, to one-tenth the distance it ought to go—while the regret is heightened by a consciousness that all our exertions, in delineating its qualities or concentrating its information, must be very inadequate to the object in view. It will be readily enough imagined that, under these circumstances, we shall adhere, with more than usual rigour, to our fundamental principle of confining our analytical labours to the most useful and practical parts of a volume embracing a great proportion of ingenious physiological and even metaphysical reasoning and inquiry.

It is proper to state, in limine, that our author has held, during the last ten years, the appointment of physician to an Institution where a great proportion of the complaints belonged to that class of maladies which are the subject of this treatise. Here a variety of phenomena have presented themselves, from time to time, to his notice, which appeared to throw light on some pathological enquiry, or to suggest some practical indication. With the hope of contributing his mite to the general stock of knowledge, respecting an interesting but obscure class of diseases, our author has modestly submitted this volume to the public, and we are convinced that, in so doing, Dr. Prichard has materially added to the edifice of his former reputation.

Our author observes in his preface, what indeed several others have lately observed, that facts which have fallen under his notice induce him to suspect that disorders of the nervous system are, in the majority of cases, secondary and sympathetic affections—often at least symptoms of some latent disease in another part of the constitution—particularly in those organs which are subservient to the natural functions. This observation led our author into the attempt to discriminate these affections into certain classes, according to the nature of the primary diseases, of which they are symptoms or indications. The arrangement which our author has adopted certainly appears very complicated, and will doubtless be objected to by fastidious critics and indolent readers; but we think he has offered substantial reasons for his plan, which appears to place the facts and analogies on which his conclusions are founded, in the clearest point of view.

The first chapter of the work, containing a "physiological survey of the functions of the nervous system," intermixed with a considerable proportion of metaphysical observations and speculations, we must pass over in a very rapid manner. We may first remark, en passant, that Dr. Prichard, while he views the brain and nervous system as the *instrument* of sensation, perception, memory, and many other intellectual operations, yet considers some of the higher faculties—judging or reasoning for example, as appertaining to mind, soul, immaterial principle, or whatever other designation we may apply to that mysterious essence. He justly observes that there is no analogy between remembering and judging—between the operations of a faculty which only recalls the impressions produced by external objects, and of one which is conversant with abstract relations, the acts of which are altogether distinct from any other class of intellectual phenomena. Our author avers that he knows of no disease of the nervous system in which the *reasoning* or the intellectual faculty is perverted. It is a common remark that lunatics reason correctly from false premises.

"If this faculty is really not subject to disease, when the brain is in a distempered state, and its functions are generally thrown into disorder, this circumstance seems to afford a presumptive proof that its exercise is independent of the brain, and belongs entirely to the mind." 25.

Dr. Prichard's divergence from materialism is still more conspicuous in the following passage, wherein the *passions* are properly attributed to the immaterial principle within us.

"I am at present acquainted with no fact, either in physiology or pathology, which furnishes any ground for presuming that those mental phænomena, which are termed passions, take place through the instrumentality of any corporeal processes whatever. It seems to me probable that they are affections of the soul, or immaterial principle, and that primarily, and without the co-operation of any part of the corporeal structure. The phænomena displayed in the viscera are merely the effects of the sympathy between the mind and the body: they are at any rate consequences, and not causes. As the organized structure acts upon the mind, in the case of sensation and perception, so, in the instance of the passions, the primary operations of the mind react upon the body. They act in different individuals upon different organs: the influence of terror in one person gives rise to violent palpitation of the heart, in another to diarrhœa." 30.

Our author makes many acute and highly interesting remarks on the passions, appetites, propensities, affections, &c. and comes to the conclusion (in which we are disposed to coincide) that even *these* are traceable to the immaterial principle, and are all resolvable into the *desire* for pleasure, and the *aversion* to pain, which desires and aversions it would be absurd to attribute to mere organized matter. In these conclusions it is evident that Dr. Prichard is directly at issue with the speculations of Gall and Spurzheim.

In respect to volition, or that conscious act preceding every voluntary movement of the muscles, there is no reason, Dr. Prichard thinks, for believing that it has any local seat in the body, or that its exercise is effected or preceded by means of any organic change. It is an act of the mind, in the performance of which the nervous system, as far as we know, has no share.

"Volition may be said to stand in the same relation to the passions and propensities, which judgment, or the rational faculty, holds with respect to the other powers of the understanding. Sensation and perception, memory and imagination, present to this highest intellectual faculty the objects on which its powers are exercised. It stands as supreme arbiter: surveys the testimony of the inferior faculties; compares, discerns, judges what is true and what false; what right and what wrong in morals; what is expedient and what inexpedient in the business of human life. In like manner, the will compares the various motives which are presented to it by the desires and aversions, the appetites, affections, and other active and moral powers, and makes its own choice from among them. It is important to observe, with respect to the theory of the mind, as well as to that of the nervous system, that these two highest powers, the principal or governing ones, in both classes of mental faculties, are, as far as we can learn, distinct from, and independent of, the organization; although the objects on which one is exercised, and the means by which the other produces its effects, are furnished by the corporeal organs.

"Volition, as we well know; even an act of the will to move the limbs; may take place in a paralytic; but it becomes abortive, unless the brain, the nerves and muscles, be in a state to obey its mandate." 39.

The second chapter of the work before us comes more immediately to the subject of the treatise, and exhibits a slight, but very interesting pathological survey of the diseases incident to the nervous system. The author justly observes that our principal or only resources in the investigation of the subject are dissection—the phenomena during life—and an attentive observance of the juvantia and lædentia. In the second section of this chapter the author gives a rapid view of the connexion of disorders of the nervous system—particularly between apoplexy, paralysis, epilepsy, mania, vertigo, tremor, somnambulism, chorea, hysteria, &c. Every practitioner knows the great family likeness that prevails among the diseases above specified—how often one precedes, succeeds, or is converted into another. Numerous instances and illustrations of these connexions, successions, and conversions are brought forward by Dr. Prichard from his own experience and the most authentic records of others, and to these we would wish to direct the attention of our junior brethren in particular.

In the second section the application of this doctrine of family likeness is made to the pathology of the diseases specified. It is observed that disorders which are so nearly allied as to be liable frequently to supersede or pass into each other, must, (as it should seem,) when they have their seat in the same structure, "depend on similar deviations from the healthy condition of the system." The predisposing and remote causes may, indeed, be various or dissimilar; but the particular condition of organic structure, from which the phenomena of the disease immediately result, is probably always similar in the same complaint. What, then, is this pathological condition? Our author shall speak for himself.

"In several of these complaints, as it is well known, the most apparent circumstance in the morbid state of the brain consists in a disproportionate circulation of blood through that organ, or in an undue accumulation in the head; whether attended with those appearances which accompany inflammatory, or what is termed increased vascular action, or merely amounting to simple congestion. As it is well known that such a state exists in several of these disorders, we are entitled, if the doctrine just laid down be well founded, to infer that a similar condition is actually present in other complaints of the same class, although the proofs of it are not so decidedly manifest. This conclusion is, however, so important, that it deserves to be more particularly examined.

"In apoplexy it is well known that the most apparent circumstance in the morbid state of the brain, consists in an excessive action of the arteries belonging to the encephalon; or, at least, in an unusual repletion and distention of the vessels; or in what is termed an increased determination to the head; often producing effusions of blood, or of serum, within the skull. If the foregoing remarks are well founded, we have from this fact reason to believe, that the immediate cause of other disorders, which, by their frequent conversions and transitions, are shown to be allied to apoplexy, consists in a deviation from the healthy condition of a similar kind, though probably very different in degree, and modified by a variety of circumstances." 68.

Our readers are aware that this doctrine approximates to, or rather that it is identical with, the pathology of nervous diseases, as set forth in the incomparable work of Dr. Parry the elder. These doctrines are strongly supported by the post-mortem as well as the living phenomena. Our author observes that, when we generalize the appearances discovered in the heads of persons who have laboured under the diseases alluded to, they are found to resolve themselves, for the most part, into the effects of inflammation and of increased vascular action—such as adhesion of parts within the cranium; effusions of serum into the cavities; distention of the vessels; abscesses; hæmorrhagic effusion into the ventricles, on the basis, and into the interstitial openings of the brain; redness of the membranes, &c.

Other species of disorganization, indeed, besides the unequivocal products of inflammation, are occasionally discovered in the encephalon, in some forms of nervous diseases, as tumours, induration, softenings of the brain, &c.

But even these are probably the consequences of disordered vascular action or inflammation, though there is no doubt that, in many cases, the irritation they produce leads to an increase of the inflammation or sanguineous congestion that gave origin to them.

The doctrine of the juvantia and lædentia leads to the same result—for if we are called upon to point out one particular indication, or principle of practice which is more generally applicable than any other to distempers of the nervous system, there could be little difficulty in concluding it to consist in the means adapted for restraining determination of blood towards the head, and diminishing the quantity of that fluid circulating through the brain. *Bleeding*, for instance, general or local, though not universally applicable to all states and periods of nervous disorders, yet is very generally serviceable at one period or other of most affections of this kind, when severe or long continued. The instances to the contrary are little more than exceptions to a general rule. Passing over a good deal of shrewd observation and keen remark, we come to the third chapter, containing a general description of epilepsy.

Our author considers it impossible to give any thing like a correct definition of epilepsy, without dividing the disease into at least two species—the convulsive and tetanic epilepsy. The first or more common form may be thus defined :—"A disease manifesting itself in sudden fits, attended with a total or partial loss of sense and consciousness, and a general convulsive agitation of the voluntary muscles." The less frequent or tetanic form is distinguished by sudden fits of coma, or loss of sense and consciousness, without convulsion, but attended with a tonic spasm of the system of voluntary muscles, the whole trunk becoming rigid and inflexible during the fit. To these two forms, Dr. P. thinks, we may add a third, where the patient, with or without the premonitory symptom of vertigo, falls down in a state of insensibility resembling profound sleep, the muscular system remaining in a completely relaxed state. This last species our author designates by the term *Leipothymia*—or more correctly, "*Epileptic Leipothymia*," in order to distinguish it from somewhat similar paroxysms falling under the description of apoplexy, from which, however, it is not always easy of discrimination.

The second section of this chapter contains an outline of the history of the disease. The symptomatology of the common form, or convulsive epilepsy, does not differ from that laid down by our best authors, and is familiar to all practitioners.

"The paroxysm of tetanoid epilepsy is similar, in some particulars, to the attack now described. The patient is seized suddenly; his limbs are stretched, and the whole trunk extended and fixed by a rigid spasm; the eyes are widely open; not reverted, but staring frightfully; the pupils contracted, and quite insensible to the stimulus of the strongest light: "Erigitur quoque penis in infantibus; in adolescentibus semen ejicitur, et sæpius urina ad magnam distantiam prorumpit."* 90.

These two forms of epilepsy, though differing in appearance, are yet closely allied in pathology. In epilepsy, as in most other diseases, there is great variety in the symptoms in different individuals. In some cases there are premonitory sensations, in others none. In some instances the attack is not accompanied with convulsions. The most unvarying symptom is the state of stupor or insensibility during the paroxysm. In a very great majority of cases this amounts to complete coma. In a few very rare cases our author and others have been able to discover some slight degree of sensibility or consciousness during the paroxysm.

It is curious that epileptic attacks happen much more frequently during sleep than in waking hours. In one remarkable case under our author, a fit scarcely ever failed to seize the patient immediately after falling asleep, even in the day time. The disease pretty equally affects males and females —nor are there any temperaments, habits, or constitutions exempt from its attacks. There are periods of life at which epilepsy is more prone to occur than at others. Infants are subject to the disease; but in them it generally arises from irritation in the bowels, and disappears with the exciting cause. The first dentition also is a critical period, but if the disease appear then, it generally goes off when the dentition is completed. Epilepsy often appears, for the first time, about the eighth, tenth, or twelfth year, in which case there is danger of its becoming habitual. Still there is a prospect of its subsiding at puberty; but if this period pass without amendment, there is little hope afterwards. If, in females, the coming forth of the catamenia sometimes assist the constitution in getting rid of the disorder, it much more frequently gives rise to it—at least there is no period of life in which females are so frequently attacked with epilepsy as the period of first menstruation. The intimate connexion between epilepsy and other nervous disorders has been observed by all practitioners.

---

* Dr. Prichard says he never noticed the ejection of either semen or urine. We have seen the *latter* very often, and we believe it is no uncommon phenomenon. Dr. Gregory, in his lectures, states that he has seen both ejections.—*Rev.*

"But," says our author, "the disorder to which, of all others, epilepsy would appear, from this and similar observations, to be most nearly allied, is mania. I believe these diseases more frequently pass into each other, and what is more to our present purpose, more frequently appear in persons related to each other by consanguinity, than any others of the same class; except those affections which are strictly considered as merely modified appearances, or as sequelæ of the same disease." 97.

*Terminations, Consequences.* In children the epileptic fit sometimes terminates fatally, without any satisfactory appearances on dissection. In more advanced years this is rarely the case, unless the paroxysms are very frequently repeated at short intervals, in which event, there is danger of life or intellect. Severe epileptic fits give occasion to paralysis, amaurosis, and other consequences resulting from lesions of the sensorium. Whether the fits are severe or not, if long continued, and especially if the intervals be short, the memory and other mental faculties generally suffer, and fatuity or idiotism is too often the result of severe and inveterate cases. Another concomitant of epilepsy is "*mania epileptica,*" resembling the delirium of phrenitis, and probably depending on a similar physical condition of the brain. This affection generally appears when the patient is expected to revive from the comatose state consequent on a severe fit—in some instances it appears without any previous fit.

"The face is flushed, and the aspect of the patient is like that of a man under intoxication; he attempts to start from bed, and run about, and on being withheld, vociferates and endeavours to overcome resistance. Sometimes an appearance of maniacal hallucination displays itself, but more generally the disorder resembles phrenitic delirium. It commonly continues one, two, or three days, during which the patient requires confinement in a straight waistcoat and then gradually subsides, and the patient returns into his previous state." 100.

*Pathology of Epilepsy.* Our author thinks, and in this he is borne out by facts, and anticipated by other writers, that "*a preternatural influx of blood into the vessels of the encephalon, or an unusual fulness in some part of the vascular system of that organ,*" is the immediate precursor and occasion of epilepsy.* The reasons on which Dr. Prichard grounds this pathological doctrine are—1st, The intimate relation which epilepsy bears to other disorders of the ner-

---

* See Fothergill, Cullen, Parry, Johnson, among the moderns; and most of the ancient physicians.

vous system, depending confessedly on determinations of blood to the sensorium. 2dly. A consideration of the circumstances which most frequently give rise to the disease, and which are calculated to occasion morbid plethora of the cerebral vessels.

"Thus epilepsy often occurs in persons who have rapidly increased in bulk and fulness of habit: in men of indolent habits, who live luxuriously: the quantity of blood in the body being excessive, a slight change in its distribution occasions excessive local plethora.

"It occurs in females who labour under suppression or retention of the catamenia, or when the flow is scanty and difficult. Such women are chiefly subject to the attacks at the periods of menstruation, when it is well known that in the defect of the natural relief of the system, a variety of morbid determinations take place, sometimes to the vessels of the stomach, occasioning violent gastrodynia with hæmatemesis; sometimes to the pulmonary vessels, giving rise to hæmoptysis; at others to the external vessels of the head, when the consequence is a profuse epistaxis: all these phænomena have been witnessed over and over again by every medical practitioner. When epileptic fits appear under the same circumstances, we have reason to believe that they arise from an analogous cause, viz. a determination to the vessels of the brain." 102.

3dly. The phenomena of the paroxysm itself indicate determination of blood to the head—as flushed and turgid countenance—pulsation of the carotids—dilated pupils—stupor during the fit, vertigo preceding, and head-ache following it. 4thly. The consequences of epilepsy lead to a similar inference. Thus we not unfrequently see instances where the brain has sustained such injury from epileptic paroxysms as to produce permanent fatuity, or palsy, or incurable deafness, or amaurotic blindness. The appearances on dissection, though various, resolve themselves, Dr. Prichard thinks, "into the evidences of inflammatory action." The most common of these appearances are a turgid state of the arachnoid vessels; sometimes a reddened condition of the cerebral substance itself; serous effusion into the cavities or on the surface of the encephalon. Tubercles are found sometimes in the brains of epileptics; "but they appear to act as occasional causes, inducing, at times, local determinations to the head."* There are, we are aware, several

---

* "Even when epilepsy," says Dr. Johnson, "is determined by organic disease in the brain itself, this organic change can only be looked upon as a *predisposing* cause. It is the derangement of balance in the *circulation and excitement alone* that can produce the immediate phecases on record, where no morbid appearance has been found in the brain after the death of an epileptic patient; and we can only account for this by supposing that such an organ as the encephalon may be so much disturbed in function sometimes, as to occasion death without leaving any perceptible traces even of vascular congestion. Dr. Prichard seems not to take into consideration the state of excitement or *irritation* which we believe always precedes the vascular turgescence of the brain in epilepsy, and which may terminate life before the vascular phenomena become apparent.

Here Dr. Prichard introduces some cases illustrative of certain portions of the foregoing text, among which are two interesting cases from that judicious and excellent physician, Dr. Laird, of Guy's Hospital, shewing the power of severe paroxysms of violent coughing in occasioning a state similar to, if not identical with, epilepsy. We shall introduce one of these cases, which is short.

" 'The first," says Dr. Laird, " was that of a gentleman, about the middle period of life, and of rather full habit of body. He became, early in the spring of last year (1820) the subject of cough, which recurred in paroxysms, and was at its commencement unattended by febrile excitement, approaching in its character to hooping-cough, which was at that period very prevalent, and which there was no certainty of his having previously had. As the paroxysms increased in severity and frequency, they were accompanied by much flushing of the countenance, and upon several occasions with entire insensibility; of short duration, however, and terminating in the expectoration of some remarkably tenacious and viscid mucus. During the state of unconsciousness the eyes were fixed, the tongue protruded from the mouth, the countenance bloated, and the pulse quick and weak. The difficulties, by which the powers of life seemed in this struggle to be sometimes nearly extinguished, appeared to arise from stricture of the glottis; in the first instance, probably of a spasmodic nature, and the danger by which he was threatened, to be apprehended from instant suffocation. Under general and local loss of blood, inhalation, and the continued exhibition of conium, ipecacuanha, and mercury, the complaint gradually gave way; and there have been since neither any symptoms of pulmonary mischief, nor any disturbance of the functions of the brain.' " 111.

We must deviate from Dr. Prichard's plan of blending the history, pathology, and treatment of *mania* with that of epilepsy, because we mean to leave over the *former* disease till our next number. We shall therefore pursue the sub-

---

nomena of epilepsy. The organic change that predisposes to this *occasional* orgasm is *always* present."—*On Derangements of the Liver, 3d Edition*.

ject of epilepsy in the present article, without questioning the propriety of our author's plan, or the kindred affinity of the two diseases.

Previously to entering on the particular history of *uterine* epilepsy,* our author makes some very judicious and interesting general remarks on regular and irregular determinations of blood to particular organs and structures of the human body. Our readers are aware that we have often endeavoured to show that the efficient cause of local determinations of blood must be sought in the vessels of the *part* itself, and not in any power of the heart or general vascular system. We find that Dr. Prichard adopts this doctrine also. "It is obvious, indeed, that the last step in the process which gives rise to the determination of blood towards a particular organ, is a dilatation of the vessels of the part." We have no doubt that this dilatation is an *active*, not a passive process, effected through the medium of the nerves. The heart has the power of active dilatation as well as contraction, and why should not the vessels have the same power? We fully agree with our able author in his theory of menstruation. He justly observes that so great a portion of the vital fluid, and so much of the energy of the female constitution is directed in a particular channel during utero-gestation, and the subsequent period of suckling, that, after these exertions have ceased, the system would be subject to a formidable train of maladies, from irregular determinations, if Nature had not supplied a supplementary resource to divert the accumulating energy of the constitution into a particular channel—the catamenial. Dr. Prichard might have added, that the wisdom of Nature is manifested by the catamenial discharges anterior to pregnancy, in order to prepare the system for the new and extraordinary wants of the uterine, and lactiferous vessels afterwards.

*History of Uterine Epilepsy.* This disorder chiefly affects young females of sanguine temperament, about the commencement of the catamenial epoch, or shortly afterwards. Occasionally it takes place at a later period of life, in accidental obstruction of the menses.

---

* Alexander of Tralles says, that epilepsy is generated in three ways, either in the brain, the stomach, or *other parts* of the body. "Morbus igitur comitialis tribus modis generatur: vel enim capite primaris laborante, vel stomacho, vel *alia quadam particula* affecta, et pravitatem in ea subsistentem, *ad caput remittente.*" This is as good pathology as we have in the present day.—*Ed.*

"In many instances the catamenia have taken place naturally for some months, when, owing to exposure to cold or damp weather, o wetting the feet, at the period of their recurrence, and while they are actually taking place, a suppression follows, and epileptic fits are the consequence. In other cases, and without any assignable cause, the flow is, at some particular period, much more scanty than usual, and of an unnatural quality; and then about the commencement, and sometimes even after the orgasm has passed over, the head becomes suddenly affected with pain, strong pulsation, vertigo, and epileptic fits ensue. In other examples, the catamenia altogether fail to make their appearance at the proper age, and the person becomes subject to disorders of the head at the same time, and to epileptic fits, which continue until the function of the uterine system displays itself, and often more or less severely during life." 149.

Often there is nothing peculiar in the character of the fits of uterine epilepsy. They sometimes commence with the aura epileptica—at others, are preceded by pain in the head, pulsation of the carotids, and vertigo—not unfrequently there is no premonitory sign. The character which has appeared to our author to belong more particularly to uterine epilepsy, is the form he has termed *leipothymia*, the description of which we have already given a few pages back. Here our author details fifteen cases of uterine epilepsy, for the sake of elucidating the history, rather than the treatment, of the disease. Of these, we shall not be able to notice more than one or two, as specimens.

*Case.* Anne Davis, ætat. 17, of sanguine temperament, was brought into the Bristol Infirmary, in a comatose state, on the 18th January, 1820. It appeared, that the catamenia suddenly disappeared about five months previously, in consequence of a severe cold. Two months after the suppression, she became affected with head-ache and giddiness, attended with shiverings. On the day of admission, these symptoms had come on with unusual violence, and increased until she became quite insensible, and had the appearance of a person in apoplexy. Twenty ounces of blood were abstracted from the arm, after which she began to recover her senses. A cathartic powder was administered. Next day, sixteen ounces of blood were taken away. A cathartic mixture, *ter in dies.* On the 20th she had quite recovered her faculties, but still complained of her head. Repeated the venesection to sixteen ounces. Our author did not see her again till the 27th, when he learnt that the catamenia had begun to flow soon after the last bleeding. They had ceased on the 26th. On the 31st, she was again attacked with headache, diarrhœa, and thirst. Venesection repeated. She was discharged cured, on the 8th, from the infirmary." 152.

We shall give a slight sketch of the 14th case, because there is a dissection disclosing some peculiarities. The patient was a girl of slender habit, fair, sanguine complexion, who had been about two years subject to epileptic fits, when she was admitted into the Infirmary on the 10th June, 1819. The fits were very irregular in the period of recurrence—sometimes coming on repeatedly in the course of a day—at other times having an interval of several days. The fits are more troublesome about the catamenial periods, the menses being scanty, and accompanied with much pain. Her aspect was imbecile. Bled and purged; and these processes repeated several times between this and the 8th July, when she was still unrelieved, and the epileptic paroxysms frequent and severe. She was ordered turpentine emulsion thrice a day, and had no fits for ten days. They then recurred as bad as ever. Blisters, caustic issues, cupping, digitalis, oil of turpentine, and various other remedies were used in vain. On the 17th of the succeeding March, she expired in a paroxysm of the usual character.

*Dissection.* A slight amber-coloured effusion on the left side, between the membranes of the encephalon—some little fluid in the ventricles and about the medulla oblongata—also in the theca vertebralis.

"The left lateral sinus, through its whole length, was filled up by a substance, very different in its nature from a recent coagulum, and apparently consisting of a deposition of lymph, which had become organized. It appeared so completely to occupy the calibre of the sinus as to have entirely impeded the transit of blood through it." P. 179.

In the thorax some fluid was effused in the cavities, and some slight derangements in the abdomen.

We observe, throughout the whole of these cases, that the main fulcrum of treatment was depletion, general or local, and that nothing else appeared to do so much good. This is a strong confirmation of the truth of our author's doctrine, respecting vascular turgescence of the head as the efficient cause of epilepsy.

*Treatment of Uterine Epilepsy.* Our author's therapeutical indications are grounded on the cases detailed, and on the general pathology of the disease. When epilepsy has supervened on the sudden disappearance of the catamenia, the consequence of exposure to cold, or other noxious causes applied during the orgasm—or when they cease to recur regularly, the patient, mean time, becoming epileptic at uncertain intervals—or, lastly, when menstruation fails to take place at the usual age, and epilepsy appears in its stead—in all these instances, Dr. Prichard thinks himself warranted in concluding that, the paroxysms indicate an attempt of the constitution to restore or set up the uterine function—in default of which, morbid congestion in the brain takes place, and the consequences of that state. Another division of uterine cases connected with *dysmenorrhœa*, will be spoken of hereafter.

"The practical indications are, first, to relieve the morbid determination to the head; secondly, to restore the natural determination to the uterine system: or, thirdly, if that cannot be done, to bring the constitution into a state in which the injurious effects of amenorrhœa are felt in a less degree." 182.

One of the most important resources we possess, for the first indication, is blood-letting—the immediate effect of which, is generally relief of the pain and oppression in the head, and a subsidence of the carotid and temporal pulsations. "Sometimes the use of the lancet is speedily followed by a restoration of the catamenia." The following observations on the effects of venesection in inflammatory affections, we shall give in the author's own words, as we cannot abridge them without detracting from their interest.

"If a person labouring under an attack of pneumonia, hepatitis, or other inflammatory disease, is freely bled, so that either absolute syncope, or a degree of relaxation approaching to syncope, be induced, one of the following effects will commonly ensue:—First, The morbid determination to the inflamed part, or the particular state of the vessels in which the disease consisted, is entirely overcome, and the disease will be found to be removed: this event ensues most frequently when venesection has been employed within a few hours after the commencement of an inflammatory disease: or, secondly, the pain, and other signs of local inflammation, still remain in the part affected, and require other efforts to remove them: or, thirdly, when the patient recovers from his fainting state, and the pulse regains its force; or sometimes, after an interval of sleep or collapse, the disorder of the part originally affected is found to have entirely subsided, but a new morbid determination to some other part of the system is discovered, sometimes of less importance, at other times endangering life in a greater degree than the original affection: and this new disease often requires a repetition of bleeding, which, in its turn, ushers in a fresh attack in some other organ.

"It is needless to adduce particular facts as exemplifications of the last assertion, as every medical practitioner must repeatedly have witnessed such phænomena. They occur most frequently in cases of inflammation of the external parts, particularly of the joints; such as are denominated rheumatic: but they are also frequent in inflammatory disorders of a different description. If a patient recently attacked by pleurisy, which is confined to one side of the thorax, is bled until syncope supervenes, the disorder often shifts to the other side. After inflammation of the peritonæum has been suddenly relieved by bleeding, the head is often attacked. In short, the migratory tendency of many inflammatory disorders is a trait extremely well known; and it is equally well known that the change of determination is very often an immediate consequence of bleeding.

"It must therefore be allowed that evacuations of blood, sufficient, with respect to quantity, to reduce the action of the heart and the circulation, have not only this immediate effect, but have also a secondary effect, viz. they give rise to a change in the distribution of blood to various parts of the vascular system. In other words, they excite new determinations, which in general shew themselves immediately after the collapse, induced by the evacuation, ceases. Now the new determinations which arise after bleeding are sometimes morbid phænomena; sometimes the restoration of natural and healthy processes. Instances of both kinds occur in the recitals of the foregoing cases. In some instances the natural determination to the uterine system, and a flow of the catamenia, followed: in others, an attack of phlegmasia or painful affections of the joints. Either event was salutary in cases of uterine epilepsy." 183-4-5.

In order to insure the best effects of venesection in uterine epilepsy, Dr. P. advises that the blood be taken away, while the patient is sitting up, until syncope begins to take place. No general rule can be laid down as to the repetitions of bleeding—these things must be left to the judgment of the practitioner. Our author cautions us, however, not to be deterred from persevering in the plan commenced, as long as the patient's strength will bear depletion, or the disease continues obstinate; for relief is not to be always looked for in the first instances—on the contrary, the fits will often recur uninterruptedly till after repeated bleedings.

As an auxiliary, the semicupium, or balneum, at 96° or 98°, is useful in producing a general relaxation of the extreme vessels, and a determination of blood into the superficial arteries. It is peculiarly apt to determine to the uterine vessels. Several of the cases detailed by our author illustrate these remarks. A very hot bath, however, should not be ventured on without due caution. While the patient is in the warm bath, the back, loins, and abdomen should be rubbed with flannels; and when put to bed, the circulation through the extreme vessels should be promoted by draughts of warm diluents. The same indication will be promoted by stimulating glysters. "An ounce of ol. terebinth. with an ounce of oleum ricini, makes an enema sufficiently powerful." Our author has no experience of blisters applied to the sacrum or pubes. The oil of turpentine, our author considers to be one of the most diffusible stimulants in the whole Materia Medica, and the most potent emmenagogue. He has ascertained by a sufficient number of experiments, that this medicine has considerable power over maniacal affection connected with defective catamenia. From half a drachm to two drachms of the oil is to be given in an emulsion thrice a day—or two drachms may be given at one dose going to bed. The black hellebore and pulvis sabinæ are not so powerful as the turpentine. Should these means prove abortive, we must endeavour to bring the constitution into a state in which the defect of the uterine function may be productive of least injury. For this purpose, it is necessary to prevent a return of the plethoric state, by observing an antiphlogistic and attenuating regimen, exercise in the country, and open bowels.

In the next place, the effect of artificial drains by issues or setons in reducing plethora, is considerable—but they have a further efficacy in uterine epilepsy. In several of the cases detailed by our author, a new morbid determination taking place in the system, suspended or removed epilepsy, where every other remedy failed—these were, phlegmatia dolens, rheumatism, cutaneous eruptions. This consideration indicates the propriety of establishing a supplementary drain or disease, as a substitute for the natural one.

"This may be done by setons in the nape of the neck: perhaps more favourably by issues or setons on the sacrum, or above the knees. If these applications are too troublesome, an issue in one or in each arm will have a similar effect, in a certain degree. If there are any constitutional disorders which may act as drains or diversions, they should be encouraged. Of this nature are cutaneous diseases, discharges from the legs, and other local maladies." 190.

Nature has yet another resource in reserve for uterine epilepsy—it is pregnancy. Even if this should not take place, our author has reason to think, that marriage would generally remove the disease.*

In cases of epilepsy dependent on *dysmenorrhœa*, the practice must be somewhat modified. *Moderate* bleedings, and those means which promote relaxation of the system, and a determination towards the uterus, are principally to be relied upon. The bleedings must not be carried to syncope, lest they should cause a total cessation of the menstrual discharge. They must be managed so as to produce a perceptible degree of relaxation in the pulse—the immediate effect of which is, generally, a relief of pain in the head, and a removal of the uterine constriction, the catamenial flow becoming free and natural. At the same time, the other means before described, are to be adapted to the exigencies of the case. "If the dysmenorrhœa should be invincible, much may be done to ob-

* See two curious cases of this kind related by Hoffman; *vol.* III. *p.* 21.

viate its injurious effects on the habit, by constant exercise, purgatives, and artificial drains."

The sixth chapter of the work is on epileptic and maniacal cases arising from metastasis of morbid action from other structures to the brain. In pursuance of our plan, we shall adhere to the subject of epilepsy, reserving mania for our next, in which we shall introduce information from other quarters besides the work before us.

The doctrine of metastasis is as old as Hippocrates; and all the difference between the opinions of the humoralists and physicians of the present day, consists in this—that the former considered metastasis a translation of peccant *matter*—the latter, a transference of morbid *action* from one place to another.

One of the most frequent and striking examples of metastasis to the head, is that which succeeds the drying up of ulcers, or the disappearance of eruptions on the surface. All authors have recorded, and all practitioners have seen frequent instances of this kind. It is also well known, that severe affections of the brain occasionally take place, after the sudden disappearance of gouty or rheumatic inflammation of the joints, and also of the serous membranes, as of the pleura and peritoneum. Another metastasis is that of dropsical inflammation to the brain, giving rise to convulsive and maniacal affections. Several cases of epilepsy supervening on the sudden disappearance or diminution of anasarcous effusion, are on record. Dissection, in such cases, generally discovers serous fluid in the cavities of the encephalon, and it has been supposed that, the affection depended simply on the absorption of the fluid in one place, and its deposition in the brain. We are by no means certain whether this be the case, or whether it be a new action set up in the head, the consequence of which is effusion there.

Tumours formed in any part of the body, require a particular current of fluid towards them for their growth and support. This, by its continuance, gives rise to a particular state of the constitution, that renders the system liable to suffer on the removal of the tumour, from the want of an accustomed drain. The effects of this metastasis are various:—sometimes epilepsy, sometimes mania, results. Cases in illustration of both events are given by our author, for which we refer to the work. Other examples of metastasis might be adduced, but it would be useless to enter into a more particular enumeration of varieties which lead to no important conclusions, pathological, or practical.

In respect to the *treatment* of metastatic affections of the head, our author thinks that, when the accident happens in consequence of the stopping of an habitual hæmorrhage, as piles or epistaxis, especially if the subject be young, then venesection may be ventured on as freely as in the uterine cases, treated of in a preceding chapter. The same observation applies to metastatic affections resulting from the healing of old ulcers, and also from translation of hydropic inflammation.

"In cases of metastasis, when the primary disease has been situated in serous membranes, the disease of the encephalon probably differs considerably from that which occurs in the forms of disease above mentioned: the marks of increased vascular action, or arterial plethora, are less decided. The theory of these cases is not so well understood as to afford a safe guidance to practice; but, as far as I can venture to draw an inference from the *facts* of which I am in possession, I believe that topical bleeding, by cupping glasses or leeches, is preferable, in such instances, to venesection in the arm: the strength of vascular action in the head and neck, and the degree of heat of the scalp, as well as in the extremities, are circumstances which must be considered in directing these measures." 239.

The same observations apply, Dr. P. thinks, to affections of the head from recessions of the exanthemata—for example, on the disappearance of measles, where large general bleedings would be improper, the circulation, in such cases, being weak and irregular. "It is only when the general circulation is strong, and the skin generally hot, that it promises advantage." Purging is a remedy which may safely be employed in cases of this description, and often answers the purpose of bleeding. The most important indication is to produce a new determination, or restore that which existed previously to the metastasis. The hot bath, pediluvia, frictions, blisters to parts formerly affected, irritating ointments or liniments, sinapisms, &c. &c. may be advantageously called into use.

"Another remedy which I have found efficacious in this class of disorders is mercury; so administered as to bring on ptyalism. In the first case reported in this chapter, the patient recovered as soon as the constitution became affected by this remedy; and I have witnessed an equally decisive effect in other instances. I am by no means disposed to approve of the frequent, much less of the indiscriminate use of mercury, in disorders of the brain and nervous system: but in cases of the description I am now referring to, I am persuaded, by experience, that it has a peculiar efficacy." 241.

The seventh chapter of the work before us in on epileptic affections depending on a disordered state of the intestinal canal.

In some preliminary remarks, our author observes that, affections of the nervous system arising from disorder of the digestive organs, have long been familiar to most medical practitioners. But they are considered, in general, to arise from what is termed sympathy with the irritated portion of bowel. It is not commonly imagined, he observes, "that any inflammatory process is set up within the cranium, in consequence of the disordered state of the digestive organs; or, that organic disease of any part of the nervous system is an intermediate step between the original malady in the abdomen, and the subsequent manifestation of its effects in the state of the animal functions."

"I am persuaded, however, after a long continued attention to this subject, that the general progress of disease, where morbid affections of the intestinal canal are followed by disorder of the nervous functions, involves an intermediate affection of the cerebral and nervous fabric itself. The proofs of this opinion I cannot bring into one connected statement: they will result from the accounts of particular cases, of dissections, and from other observations, which are to be comprised in this chapter. The disease which I supposed to be produced in the brain, and other parts of the nervous system, is a state of morbid plethora in the blood-vessels belonging to that fabric. I shall not pretend to determine whether this always constitutes a degree of inflammatory affection, or sometimes amounts only to simple congestion. I have indeed before observed, that I do not know in what consists the difference between these states." 243.*

Our readers are aware, that we have all along impressed on the minds of our junior brethren, the importance of remembering that sympathetical functional disorder will, if long continued, end in organic disease, and this, we believe, is the simple meaning of Dr. Prichard's pathology. We can scarcely believe that our author wishes it to be understood, that he supposes inflammatory action to be the very first effect of sympathetic influence radiating from distant parts on the sensorium. We doubt not, indeed, that some peculiar condition of the vascular system of the brain, may be connected with this functional disorder; but we imagine that we are very far from being authorized to pronounce this condition—inflammation.

Although we are ignorant by what train or connexion this disorder supervenes on the primary affection of the intes-

---

* We refer to our analysis of Dr. Parry's invaluable work for precisely the same doctrine. Speaking of the *cause* of nervous affections, Dr. Parry remarks that—"this cause is excessive impetus of blood acting on the medullary substance of the brain, or some other part of the encephalon." 292. Indeed, we have to complain of Dr. Prichard for not more frequently noticing Dr. Parry's doctrines. We have now, however, put it out of the power of authors to slur over the sentiments of this illustrious pathologist.

tinal canal, yet it seems to be by a different process from that which subsists in the case of metastasis—in which the disorder of the new part takes the place of the old, which subsides. Our author has reason to believe that the disorder of the intestinal canal itself is much more frequently of an inflammatory nature than is generally suspected.

"In that state of the canal which gives rise to costiveness, alternating with diarrhœa, and accompanied with indigestion, flatulence, and eructations, anorexia, and nausea, transient but often acute pains in the hypochondria, livid and yellow suffusions of the skin, viscid secretions in the mouth, white furred tongue, redness of the fauces and palate, the whole train of symptoms often depend upon a low degree of chronic inflammation in the mucous membrane of the intestinal canal: and this is, perhaps, a frequent, if not an ordinary state, in cases where severe nervous disorders supervene on complaints of the stomach and bowels. In some of the following cases this was certainly the condition of the disordered parts." 245.

We believe that the above pathology is nearly correct, excepting that we think our author loses sight too much of that state called *irritation*.

*Enteric Epilepsy.* Epileptic fits are sometimes obviously connected with the presence of irritating matters in the stomach and bowels; and accordingly this modification of the disease has been long recognized by medical authors. Our author thinks that epilepsy has been too often considered as an idiopathic affection of the brain, nor does he think it has ever occurred to any one to consider this disease, "in the majority of instances, symptomatic of disorder in the natural functions, or of ailments in which the brain only participates in a secondary way."* Our author observes, that

---

* We think this observation hardly warranted after perusing the following extract from a work published four years ago, and widely circulated.

"Our attention is to be particularly directed to the organ or part which is the *common or general seat of the epileptic irritation*, and from whence, at the moment of attack, the said irritation seems to dart in an instant to the sensorium, and there, by deranging the balance of the excitement and circulation, to produce the phenomena of the disease. This seat of irritation may be, and often is, in the coverings, or even substance of the brain itself, where, however, it only *occasionally*, as in the other instances, disturbs the vital functions of this organ. *More frequently* the domicile of the mischief is at a distance—especially in the digestive organs. In this case the premonitory symptoms are clearly marked, as pain in the stomach, tension in the epigastric region—loss of appetite, &c. When from worms, the patient, during the seizure, presses the abdomen with his hand. Indeed, when children are affected when the disease has its domicile at a distance from the brain, it is only to be cured by removing the primary cause, and not by the exhibition of a " set of medicines supposed to be possessed of certain anti-epileptic powers." But we may remark on this passage that, as these anti-epileptic powers of certain medicines (say for instance the argenti nitras) were found out by experience, not reasoning, it is hardly fair or prudent to reject them from our practice, because their operation cannot be rationally explained upon any of our now known principles. How do we know, in fact, whether the argenti nitras does not sometimes cure or mitigate epilepsy, by establishing a new or removing an old irritation. If lunar caustic be applied to a sound part, it will cause irritation and ulceration—if to an irritable and disordered surface, it will lessen pain and irritability. Is there not ground for supposing, then, that this remedy acts on the principle so much contended for by Dr. Prichard himself?

When enteric epilepsy, though at first the result of gastric or intestinal irritation, has taken a firm hold on the system, through the influence of habit, or by the effect of disorganization, " occasioned by long continued morbid action," it then becomes a permanent malady.

" In other instances this disease may be cured by a plan of treatment directed to the removal of the morbific circumstances which occasion it. The longer the duration of the disease has been, the less prospect is there of entirely overcoming it: still, if the disorder in the abdominal functions is within the reach of medicine, the case is not, after any period of time, altogether desperate. Nature sometimes effects a cure after a patient has been many years subject to the recurrence of fits, and even after the brain has manifestly sustained much injury." 253.

It is well known that epileptic fits often occur in connexion with the presence of worms in the intestinal canal, both of children and grown persons. The tape-worm, in particular, frequently induces epilepsy. The enteric form of the disease occurs at all periods of life. During dentition,

---

with epileptic fits, without any previous frights or other ostensible cause, and who, in the mean time, have pale complexions, dull eyes, dilated pupils, clayey stools—tumid abdomens, languid gait, &c. there is scarcely a doubt but that worms are the exciting cause.

" The biliary organ is very often the seat of the primary epileptic irritation, which is evinced by pain in the region of the liver—deranged intestinal secretions—borborygmi, and a yellowish suffusion previous or subsequent to the paroxysm. Even a morbid secretion of bile itself, as was observed by Hippocrates, will determine epilepsy." *Johnson on the Influence of the Atmosphere, 2d Ed. p.* 102-3.

when the constitution is much disturbed, the bowels often fall into an irregular state, the precursor of convulsive paroxysms. These disorders quickly subside under a proper regimen, but are very liable to return, when the primæ viæ are suffered to fall into a distempered state.

There is nothing very peculiar in the fits of enteric epilepsy—being sometimes accompanied with convulsions, at others bearing the character of leipothymic paroxysms. The symptoms of disorder in the functions of the stomach and intestinal canal are not so strongly marked in this disease as in enteric mania, but still there is a manifest deviation from the state of health. The bowels are constipated, and often require enormous doses of purgative medicines. In other cases constipation alternates with diarrhœa. The evacuations are generally of an unhealthy appearance, and display a disordered state of the gastric, intestinal, and hepatic secretions—in short, "an imperfect action of the digestive structure." The appetite is variable—often there is an unnatural appetite approaching to pica—a characteristic of enteric epilepsy as well as mania.

"I apprehend that the pathology of this disease is very similar to that of enteric mania. The distempered state of the intestinal canal is the same in both disorders: according to the different morbid predispositions of the nervous system, the same exciting cause or irritation in a remote part of the constitution, gives rise in the brain of one person to a disease which manifests itself in maniacal impressions; and in another individual occasions a differently modified disorder in the state of the same organs, the symptoms of which are attacks of epilepsy." 257.

*Treatment.* This is somewhat different from that employed in uterine and metastatic epilepsy. In the former cases our object was to alter or restore determinations of blood—in the present case our chief indication will evidently be to remove the disorder of the alimentary canal, the fundamental cause of the disease in the brain. In the mean time, it is necessary to relieve the secondary but more urgent affection of the sensorium, which is often manifestly loaded with blood. It is still more evident that we are to direct our attention to this point, when there is a constant stupor, drowsiness, head-ache, dilatation of the pupils, vertigo, starting from sleep, or agitating dreams. Here we are authorized to bleed generally and locally, apply cold to the shaven scalp, and blisters to the nape of the neck. While the threatening symptoms are in process of being removed or mitigated by measures of this kind, we are not to neglect the bowels—indeed, in a great many instances, our sole attention may be directed at once to this object. If there be evidence of a loaded state of the stomach and bowels, emetics and purgatives must be given. "It is often proper, says our author, to begin by prescribing five or six grains of calomel, with one, two, or three of tartarized antimony. This mixture will often excite vomiting and purging at the same time. If necessary, it may be followed by a dose of ipecacuanha, to promote the former action." If it fail to act on the bowels a cathartic may be given.* Our author has succeeded in obtaining relief by injections of an ounce or two of ol. terebinthinæ with an equal quantity of castor oil, mixed with gruel. The use of the warm bath promotes the relaxation of the bowels, and contributes to relieve the system in other respects.

The primæ viæ being thoroughly cleared, and the acute cerebral affections reduced, then a more continued treatment is required in the disease, considered as a chronic malady. In these protracted cases a long continued diarrhœa kept up by purgative medicines, reduces the strength of the patient, while at the same time a morbid secretion in the bowels feeds irritation of the system, and sustains the symptomatic disease. After clearing the primæ viæ therefore by calomel and other means, a steady and regular action ought to be kept up in the bowels by stimulating laxatives. The compound decoction of aloes may be given three times a day, with half a drachm of carbonate of soda. The mercurial and compound aloetic pill may be taken; or the blue pill with assafœtida and extract of colocynth, alternated with salts and senna.

Of all remedies which our author has tried in epilepsy, he found none so frequently useful as oil of turpentine. He has exhibited it in many cases of intestinal disorder, and observed that it was most serviceable when given in pretty large doses, as from half a drachm to two or three drachms three times in the day. Even in the latter quantity it can be very well borne when given in the form of an emulsion carefully prepared, by diffusing the oil, by means of honey or mucilage in some strong aromatic water. The modus operandi of the oil our author cannot pretend to explain, but he has found, from the experience of many hundred cases, that it very soon changes materially the state of the intestinal canal.

"It occasions moderate and regular evacuations, corrects the tendency to a frequent repetition of griping and irritating stools, and relieves, or completely removes flatulence. At the same time the

---

* "The combination," says Dr. Prichard, "of calomel and tartarized antimony acts more easily and speedily on the stomach than the latter salt given alone."

oil of turpentine exerts a peculiar sedative or tranquillizing power on the nervous system. It lessens irritability, the disposition to starting and convulsive twitching of the muscular fibres, and promotes sleep." 263.

If it occasion a troublesome vertigo or nausea, when taken in the day, a double dose may be given at bed time. The best vehicle for the emulsion above alluded to, is milk—half an ounce of the former to a tea-cupful of the latter. By a persevering employment of this medicine, combined with some auxiliary measures to be noticed farther on, Dr. P. has frequently known disorders of this class relieved, and sometimes removed, which seemed at first hopeless.

Our author apprehends that the nitrate of silver has been found chiefly useful in cases of enteric epilepsy, as this medicine, as well as several other metallic salts, for instance, the sulphate and oxyde of zinc, &c. possesses a certain efficacy in many disorders of the stomach, connected with pains of the head. This may be so; but we have seen the nitrate of silver produce much benefit in epileptic subjects where there was no evidence of gastric or enteric derangement whatever. Our author's experience has not led him to place any great reliance upon this remedy, or upon any of the same class, in epilepsy—"though it has appeared to mitigate the disease in some instances, or to render the fits less frequent." In chorea he has found it the most useful of the metallic salts. It is principally in enteric epilepsy that antispasmodics have acquired reputation, as they allay many troublesome sensations and flatulencies with which the patient is oppressed. The best forms, he observes, in which they can be given, are the spiritus ammoniæ fœtidus in camphor julep—pills of assafœtida with oxide of zinc—tincture of assafœtida—ammoniated tincture of valerian—compound galbanum pill, &c. &c. The doses should be frequently repeated. Sometimes glysters of assafœtida with oil of turpentine have a good effect.

Whatever class of alteratives we may employ with the view of restoring a healthy state of the gastric and intestinal secretions, their efficacy will be greatly promoted by occasional brisk purgatives—say every third or fourth day—and sometimes emetics. Warm clothing, the warm bath, and abdominal frictions, are auxiliaries. The diet, of course, is to be restricted, and all vinous and fermented liquors proscribed.

In some cases of enteric epilepsy, there is obstinate and almost indomitable torpor of the bowels. Here powerful cathartics only exhaust the excitability of the intestines, and augment the disease.

"One of the principal resources we have under these circumstances is the constant use of mild enemas, which act rather as diluents than as stimulants. A large quantity of water should be injected into the rectum every day, or twice in the day, viz. every morning and evening. Many persons prefer warm water, and obtain from the use of it a sufficient relief. In other instances cold water has been found more effectual. I attended a man about two years ago who had a variety of troublesome disorders, the effect of habitual costiveness. This person now enjoys good health, and a greater degree of vigour than he has experienced for many years. His remedy is an enema of water, which he injects always as cold as he can procure it every morning, by means of a leather bag. He first injects two quarts, which speedily excite his bowels to evacuate their contents, and he then immediately repeats the operation." 267.

Sometimes small doses of neutral salts largely diluted, will answer in these cases, if taken early in the morning, and some walking or riding exercise used afterwards. Our author has found the following formula one of the most useful aperients, where such medicines are habitually necessary:—infus. sennæ ʒj. ad ℥ij. aquæ menthæ pip. ℥iv. infus. calumb. ℥ij. magnes. sulphat. ʒj. m. capiat cochl. iij. mane quotidie.

There are cases of enteric epilepsy, where determinations to the head will occur in despite of all medicines directed to the intestinal canal. Here we must deplete locally, from time to time, in order to save the encephalon from the effects of plethora—and, in very urgent cases, we must open the temporal artery or jugular vein. In children, leeches are the best local evacuants. Seventeen detailed cases follow, illustrating the positions, doctrines, and practices, laid down in the text. These must be perused in the original.

*Hepatic Epilepsy.* It has happened to our author to observe several cases of epilepsy, in which there were evident symptoms of active inflammation or of chronic disease in some of the larger abdominal viscera, especially the liver. In these instances he also remarked that remedies adapted to relieve the disorder of the abdominal viscera, if successful, removed at the same time, or greatly alleviated the affection of the nervous system.

"From these observations it must be inferred that there is some sympathy, or connexion depending on circumstances unexplained by any principles in pathology, between that morbid state of the brain which gives rise to epilepsy, and a diseased state of the liver, and other large viscera of the abdomen.*"

---

"* A very well marked case of epilepsy, in connexion with extensive disease of the liver, pancreas, spleen, stomach, and mesentery, is de-

Six cases are related in illustration of hepatic epilepsy, for the particulars of which we must refer to the work itself.

The ninth chapter embraces cases of cerebral disease, giving rise to epilepsy, and occasioned by the direct operation of noxious agents on the brain and nervous system. These agents are divided by our author into three classes.—1st. Mechanical injuries—2dly. Physical agents, as opium, alkohol, &c.—3dly. Violent emotions, passions, and long continued mental anxieties. Cases exemplifying each of these causes are detailed, after which, Dr. Prichard proceeds to the consideration of the treatment proper in this class of epileptic affections.

It is reasonable to suppose that the treatment of nervous affections, arising from causes which act immediately on the brain and its appendages, is more simple, and the indications clearer, than where these disorders depend on, or are complicated with, morbid conditions of other organs and functions—the main object here, in fact, is to relieve local determinations to the head, paying due regard to the general health.

In a great majority of cases, Dr. Prichard properly observes the excessive arterial action in the head is co-existent with over-excitement of the constitution in general—the usual effect ensuing from too much stimulation by ardent spirits or other exciting causes. But it is important to bear in mind that morbid determinations to the head, as well as to other parts, often co-exist with very defective vigour in the general state of the circulation, and with an exhausted or debilitated constitution—circumstances that demand a considerable modification of the treatment necessary in the former case.

"In the latter case attention should be paid to preserving and promoting the vigour of the constitution in general, at the same time that topical evacuations, and other means of relieving congestion in the brain, are adopted." 364.

Mental emotions are well known to produce great dis-

---

tailed in the 10th vol. of the Edinburgh Medical and Surgical Journal, by Mr. Clifton In this case there was a severe pain from the shoulder to the elbow of each arm, which lasted an hour before the paroxysm. I do not remember to have seen this symptom mentioned by any medical author, but I once attended a patient, who laboured several months under symptoms of hydrocephalus, in whose case a violent pain in one arm, similarly situated, was a most distressing subject of complaint. On examining the body, tumours as large as a small nutmeg, and marks of inflammation, were found in the cerebellum and in the liver: the cerebral ventricles were full of serum." 324.

turbance both in the vascular and nervous systems. Their effects, however, are more conspicuous in mania than in epilepsy, and will be more fully discussed in our next number. We must now draw our present article to a conclusion, by taking some notice of the seventh chapter on "convulsive tremor."

In some of the preceding sections our author took occasion to mention several cases in which fits of rigor or tremor seemed, on some occasions, to occupy the place of the epileptic paroxysm—particularly in those instances where the original disease had become mitigated by medicine or other means.

"I have had under my care several patients who laboured under a disease, consisting in occasional attacks of this description, in which the tremulous agitation of the muscles was so violent, and accompanied with such unpleasant internal sensations, as to occasion considerable alarm to the sufferer. These paroxysms are generally unattended with any sense of chilliness, approaching to the coldness of rigor; though sometimes the extremities, particularly the legs and feet, are cold; while the head, neck, and chest, are hot, and smoke with a profuse transpiration: the head is sometimes affected with vertigo and stupor, and sometimes with violent pain. After the paroxysm has continued some time, it subsides spontaneously, and is not followed by any accession of heat." 393.

This disease has been little noticed by authors. Tulpius and Bonetus, however, have described it. As the following case, which occurred at St. Peter's hospital, offers a good specimen of the disease, we shall insert it here.

"John Pugh, æt. 45. March 1, 1820. A carpenter, of meagre habit, low stature, dark hair. About a month ago he complained of cynanche tonsillaris; soon after he was affected in his chest; his symptoms were considered as asthmatical; his bowels were constipated. He has complained, for some time, of head-ache. On the 23d of February he was attacked, in the forenoon, by a violent tremor, which continued two or three hours, and then went off, after he had taken an emetic. It recurred again on the following day at the same time, and on every succeeding day about the same hour. He is now labouring under a paroxysm.

"On first looking at this man I should have supposed him to be under a severe rigor of intermittent fever; but, on a closer inspection, his affection appeared very different. The whole muscles of the upper extremities, including those connected with the ribs, clavicle, and scapula, were constantly agitated by a convulsive movement, which was wholly, or very nearly, confined to them. The lower extremities were quite free. The man is perfectly conscious, and able to answer any question distinctly. His pulse is quick, and appears to be irregular; but it is very difficult to feel it on account of the constant agitation of the tendons. The skin is warm, and he does not appear to have any sensation of chilliness. The upper part of the body is in a state of profuse perspiration, and smokes. He complains of vertigo and head-ache.

"I ordered him to be bled, and a large orifice was made in the arm, from whence the blood flowed in a full stream. Thirty-eight ounces, avoirdupoise, had flowed, before syncope came on. When about half the quantity had flowed, the tremor became more general, and the convulsive jerking motion now occupied the glutei, which threw him up from his seat, with the action of a man sitting on a trotting horse. As soon as he became sick and fainting, the arm was tied up, and he was laid upon a bed: the tremor immediately ceased, except some slight and partial quivering.

"He was ordered—

Pulv. Cath. ʒss. statim.

Mist. Cath. 4. qq. h.

Pil. Cath. omni nocte.

5. 11 A.M. Return of the tremor.
Cold affusion.

The cold water was thrown over him, and produced an immediate cessation of the tremor. He became afterwards hot.

"9. No return of the tremor.

Hyd. Subm. gr. v. o. n.
Magnes. Sulphat. o. m.
H. Salin. 4 hor.

"11. Tremor came on about six A. M. but continued only twenty minutes.

"He got rid of the tremors after this time, but fell into a state of debility.

"*April* 11. Appetite fails. Coughs and spits much.
Dec. Cinchon. c. Acido Sulph. ter indies.
Pil. Cath. o. n.
Mist. Aper. o. m.

"*May* 12. Recovered from his complaint. Has, however, a disorder in the arm, the effect of inflammation in a vein, which came on after bleeding. On account of this he is now in the surgery ward." 396.

The treatment of this disorder will be conducted on the same principles which guide us in epileptic and convulsive diseases in general. In most instances, Dr. Prichard thinks, it will be found symptomatic of uterine irregularity, or disorder of the digestive organs, and, of course, will be best managed in the manner recommended for those varieties.

Our readers will have remarked that we have obtruded very few of our own observations in the course of this analysis—a plan, we believe, too much neglected in general, but which should be very strictly pursued in *analytical* reviews —otherwise they cease to be such, and become mere vehicles for anonymous criticism or pedantry. We may be permitted, however, to make a few, and they will be very few, commentaries on the work before us, or rather on that portion of it which we have selected for this article.

In the first place we may observe that there is little that is new, either in the arrangement, pathology, or treatment of the disease. Almost all authors who have written on the disease have described the sympathetic species, though Dr. Prichard perhaps considers this division as being much more extensive than it is represented by others. We have shewn that, the pathology of the disease adopted by Dr. Prichard, is that of Dr. Parry, who published several years ago. The *systematic* treatment flows from the pathology, as a matter of course, and mainly consists in reducing and preventing cerebral plethora, as Dr. Parry and the best modern practitioners have inculcated.

We have been a little surprized to observe that, while Dr. Prichard traces a great deal of epilepsy, in females, to the uterine system, he should entirely overlook the influence of the genital organs, in producing the disease in the other sex. We are not solitary in believing, that irritation and torpor, (for extremes meet,) in this class of organs, determine the epileptic state very frequently; and, that medicines, which act with something like specific effect on these organs, are very useful items in the methodus medendi.

"Les organes de la reproduction," says Jourdan, "sont aussi le siége sur lequel s'exerce la cause epileptique, et d'ou, comme par irradiation partent les premiers phenomènes de l'accès. Cette espece, qu'on peut appeler *genitale*, est plus frequente chez les femmes. L'onanisme a frequemment predisposé à cette terrible maladie, &c." Our author, however, may plead that he has rather stated what he has seen, than combined the experience of others, in the work under review. But in a *treatise*, it is incumbent on an author to avail himself of the observations of others, else it becomes an essay and not a treatise.

While we perfectly agree with our author in the systematic and rational treatment of epilepsy, we are disposed to think, that we are not so far advanced in the pathology of the disease, as to renounce the aid of certain remedies to which the term *empirical* may probably apply. The nitrate of silver, and the misletoe of the oak have unquestionably been productive of service in epilepsy—and we are not authorized to object to their use, because we cannot explain their operation. We have, within these few years, given the nitrate of silver in five cases with considerable benefit—in two, with complete success. In these two cases, the medicine was continued for three months, and taken, ultimately, in doses of eight grains a day in one, and six grains a day in the other. In some other cases, the medicine produced little or no benefit. The tinctura lyttæ, carried to the effect of producing strangury, was beneficial in several cases under our observation, and we have lately exhibited it in conjunction with the argenti nitras. We may here observe, that we have seen or heard of no case with one exception, where the argenti nitras produced discoloration of the skin under five or six months, and the usual precaution given by the best practitioners, now is, not to persevere longer than three months in the use of the remedy. We have recently heard that a gentleman became blue in somewhat less than three months. This must render us still more cautious than heretofore.

We part from our author, for the present, with feelings of great respect for the talent, observation, and sound judgment, every where displayed in the work—which is unquestionably the best in the English language, on the subject of epileptic disorders. In our next number, we shall finish our review of the volume by taking up the important subject of maniacal affections.

